# Inertial Sensing Based Assessment Methods to Quantify the Effectiveness of Post-Stroke Rehabilitation

**DOI:** 10.3390/s150716196

**Published:** 2015-07-06

**Authors:** Hsin-Ta Li, Jheng-Jie Huang, Chien-Wen Pan, Heng-I. Chi, Min-Chun Pan

**Affiliations:** 1 Graduate Institute of Biomedical Engineering, National Central University, Jhongli 320, Taiwan; E-Mails: winterfrost1143@gmail.com (H.-T.L.); d0785208@hotmail.com (J.-J.H.); nocilole@hotmail.com (H.-I.C.); 2 Hsinchu Air Base, Hsinchu City 300, Taiwan; E-Mail: kkpan535634@gmail.com; 3 Department of Mechanical Engineering, National Central University, Jhongli 320, Taiwan

**Keywords:** inertial measurement, stroke rehabilitation evaluation, quantitative assessment scale

## Abstract

In clinical settings, traditional stroke rehabilitation evaluation methods are subjectively scored by occupational therapists, and the assessment results vary individually. To address this issue, this study aims to develop a stroke rehabilitation assessment system by using inertial measurement units. The inertial signals from the upper extremities were acquired, from which three quantitative indicators were extracted to reflect rehabilitation performance during stroke patients’ movement examination, *i.e.*, shoulder flexion. Both healthy adults and stroke patients were recruited to correlate the proposed quantitative evaluation indices and traditional rehab assessment scales. Especially, as a unique feature of the study the weight for each of three evaluation indicators was estimated by the least squares method. The quantitative results demonstrate the proposed method accurately reflects patients’ recovery from pre-rehabilitation, and confirm the feasibility of applying inertial signals to evaluate rehab performance through feature extraction. The implemented assessment scheme appears to have the potential to overcome some shortcomings of traditional assessment methods and indicates rehab performance correctly.

## 1. Introduction

According to the World Health Organization (WHO) stroke was the World’s second leading cause of death [1]. About 17 million people suffered strokes in 2010 [2] and three-fourths of the survivors were affected by the stroke and became disabled [3] due to the deteriorated cerebrovascular function. Research has showed that repetitive exercise training may benefit improving patients’ daily movement [4] and help patients recover, therefore, rehabilitation treatments are indispensable for the stroke patient.

In the clinic, classical assessment methods typically include the Fugl-Meyer Assessment (FMA) [5], Upper Extremity Performance Evaluation Test for the Elderly (TEMPA) [6], Wolf Motor Function Test (WMFT) [7], and other Likert-type assessment scales. Although traditional assessment scales have been used for many years and the evaluation results have been widely accepted in various fields, they are scored subjectively by occupational therapists. The variations in assessment results directly depend on the individual. Furthermore, Likert-type scales give scale scores based on numerical ranges. Thus, an individual case’s score between two scales may be an inaccurate way to describe the rehabilitation result. It thus seems that classical assessment methods may be lacking an objective standard for evaluating the effectiveness of stroke rehabilitation.

The development of objective and quantitative rehabilitation treatment assessment methods to address these issues is a non-trivial problem. Allin *et al.* [8] applied cameras to track the upper limb motion of stroke patients during desktop activities. Reinkensmeyer *et al.* [9] presented a web-based therapy system using force-feedback gaming devices to direct a therapy program, mechanically assist in the movement, and track improvements in movement ability. Further, in the effort of training the stroke patient Goffredo *et al.* [10] developed and employed a neural controller to drive a synthetic arm; Woodbury *et al.* [11] designed specific rehab tasks to enhance post-stroke upper extremity reach and function.

Recently, inertial sensors have been applied extensively for rehab engineering. Evidences showed that inertial sensors are able to provide accurately quantified human motion information although varied measurement and motion reconstruction issues need to be coped with [12]. For instance, Zheng *et al.* [13] proposed the use of position-sensing technology such as pedometers, goniometers, pressure sensors and inertial sensors, and suggested that it is feasible to build a home-based tele-rehabilitation system for sensing and tracking the motion of stroke patients. Kim *et al.* [14] proposed a wearable upper limb motion tracking method for stroke rehabilitation therapy at home, which consists of two IMUs placed on the wrist and the elbow. This method estimates the position of the forearm and upper arm by using an inertial tracking algorithm and a kinematic model. Zhou *et al.* [15] presented an inertial sensor-based monitoring system for measuring upper limb movements in real time. Kinematic models were built to estimate upper limb motion in 3-D, based on the inertial measurements of the wrist motion. To measure motor impairment of the upper extremities, Thrane *et al.* [16] proposed and calculated the arm movement ratio (ARM), the ratio of arm use duration between the more and less affected arm. Their study showed FMA of the more affected arm was strongly associated with AMR.

To reflect clinical needs, this study focuses on the implementation of an objective assessment system for the recovery of stroke patient’s upper extremities. To this end inertial measurement units (IMUs) were employed to construct a stroke rehab assessment system. We intended to reveal the recovery condition through the score calculated by three extracted indicators following examination of the stroke patient’s movement. The weight for each of the three indicators was decided by the least squares method that correlates the objective indicators and the subjective WMFT scale scores. The data from eleven recruited stroke patients and one healthy adult were used to validate the proposed assessment system.

## 2. Inertial Measurement System

**Figure 1:**
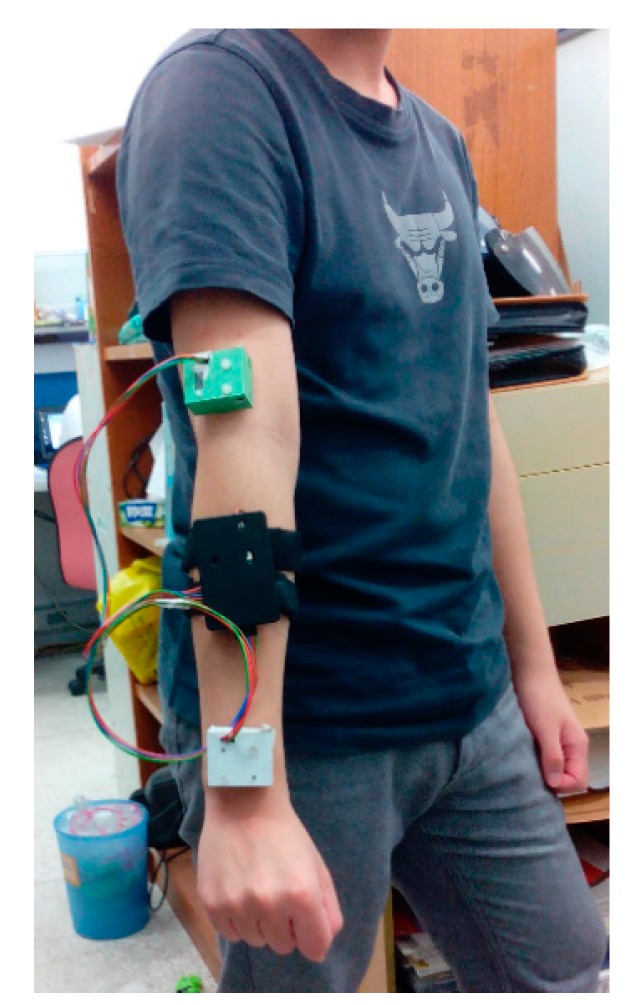
Implemented two-channel wireless IMU system mounted on the upper extremity of a healthy adult.

In the study a two-channel wireless IMU system was implemented and coded, which consists of two inertial sensors, *i.e.*, MPU-6050 Evaluation Board (EVB, InvenSense Inc., San Jose, CA, USA) comprising an MPU-6050 module (InvenSense Inc., a 3-axis gyroscope and a 3-axis accelerometer) and a three-axis magnetometer (Asahi Kasei Corp., Tokyo, Japan), an Xbee^®^ wireless transmission module (Digi International, Minnetonka, MN, USA), and an Arduino Fio control module (SparkFun Electronics, Niwot, CO, USA). Figure 1 shows the set of measuring devices mounted on the upper extremity of a healthy adult, where two MPU-6050 EVBs are packed in green and a white boxes, respectively, and the others, including an Arduino Fio, a battery and an XBee wireless transmitter are stored in the black box. A coded LabVIEW^®^ (National Instruments Corp., Austin, TX, USA) user interface was used to acquire, display and storage measurement data, calibrate the IMUs, and compute the indicators that reflect rehab conditions. The sampling rate is here set to 50 Hz, the full scale ranges of the accelerometer and the gyroscope are ±4 G and ±2000 dps, respectively, for the measurement of limb motion.

The IMUs were applied to acquire the inertial signals of stroke patients during the motion tasks before and after rehabilitation. In order to assess treatment results, measured inertial data were used to extract significant features and then quantify the effectiveness of post-stroke rehabilitation. The following sections will describe the details of assessment method.

## 3. Post-Stroke Rehabilitation Assessment Method

### 3.1. Experiment Protocol

Clinically, after rehabilitation treatment for stroke patients, therapists usually design specific testing movements such as shoulder flexion and elbow flexion, of which are simpler than rehab training or daily upper-limb movements. As a result, this study takes shoulder flexion as a movement for the assessment of rehab effectiveness. The complete shoulder flexion motion can be depicted as follows.

The initial position involves the hand hanging down with palm backward naturally. When motion occurs, the arm is lifted straight up approximately 180° until the arm is directly overhead, and it ends with the arm dropped down to the initial position. In this movement the arm is lifted vertically in front of the body in a forward direction.

**Figure 2:**
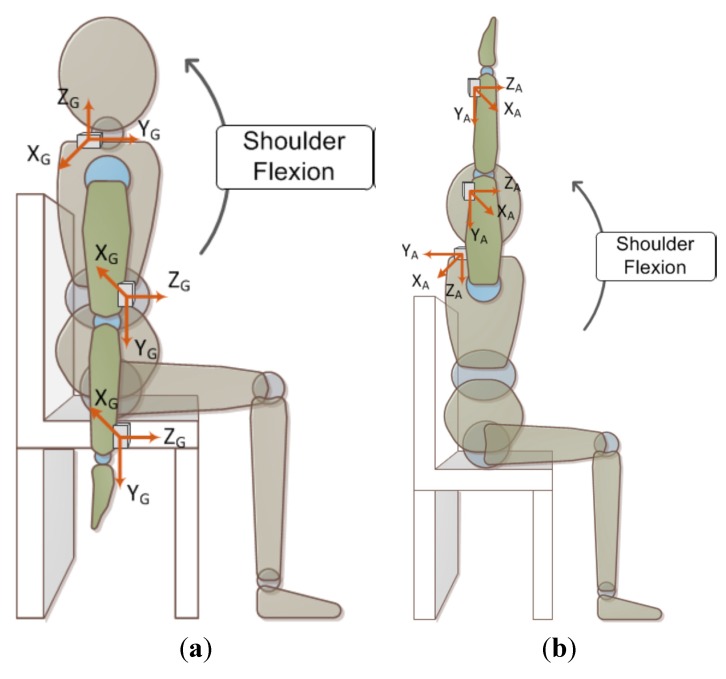
Illustration of sensors’ placement and assessment motion. (**a**) The initial position of shoulder flexion and motion direction, and the 3-axis directions of gyro marked by the subscript “G”; (**b**) The end position of shoulder flexion, and the 3-axis directions of accelerometer marked by the subscript “A”.

During the assessment, the subjects were required to sit on a chair and lift their arm as high as possible. In this experiment, the patients need repeat the movement 80 times with two minutes rest for every ten motion repeats. Figure 2 shows the positions of the mounted IMUs in the experiments, where the subject’s wrist, elbow and shoulder were equipped with an IMU, respectively. It should be noted that the coordinate systems of the accelerometer and gyro are defined differently for the data acquisition and subsequent computation.

### 3.2. Traditional Assessment Method

To evaluate the effectiveness of rehabilitation, a therapist generally gives scores based on a patient’s movement status; that is, when the patient performs the specified test movement, the therapist observes it and gives a corresponding assessment with a score. Likert-type scales [5,6,7] were thus developed for the purpose. The WMFT scale is one such Likert-type scale, that ranges from 0 to 5 which makes the WMFT characterize only a rough movement status. Additionally, these traditional assessment scales produce subjective judgments that vary depending upon individual therapists’ opinions. In order to develop an objective assessment scheme, in this work three quantitative indicators for clinical evaluation were proposed and extracted after consultations with clinicians:

(1) Elevation angle—the closer to the unaffected side, the better it is.
(2) Synergy—the less unnecessary actions, the better it is.
(3) Movement performing speed—the closer to the unaffected side, the better it is.

Some more interpretations are addressed in the next section.

### 3.3. Objective and Quantitative Assessment Indicators

The WOLF motor function test comprises seventeen tasks for affected and unaffected upper extremities, respectively. To associate with the shoulder flexion movement, six tasks were selected from the WOLF test including: (i) forearm to table; (ii) forearm to box; (iii) hand to table; (iv) hand to box; (v) lift can and (vi) lift basket; their corresponding scores were employed as an ensemble to correlate the calculated indicators for the decision of their weights. The following defines the indicators and addresses their corresponding clinical evaluation.

#### 3.3.1. Elevation Angle

For a normal shoulder flexion from the initial position to the end position, as Figure 2b depicts that the IMU is mounted on the wrist, its (local) Y-axis acceleration characterized the gravitational acceleration in the direction of the upper extremity. Thus, the Y-axis acceleration varies from −1 G to +1 G and actually follows the elevation angle of the upper extremity. Based on this observation, the indicator for the elevation angle can be defined as the ratio of maximum Y-axis acceleration on the wrist of affected to unaffected side, *i.e.*:

(1) $$A_{i,r}=\frac{A_{iy,max}+1}{A_{iyn,max}+1}\;,\;0\leq A_{i,r}\leq 1$$

where $A_{iy,max}$ and $A_{iyn,max}$ are the maximum Y-axis acceleration of the affected and unaffected sides, respectively, the subscript “*i*” denotes the sensor on the wrist *(i* = 1) or elbow (*i* = 2). It is noted that the $A_{iy,max}$ and $A_{iyn,max}$ are given in gravity (G) units. A larger calculated $A_{i,r}$ implies the elevation angle of the affected side is closer to that of the unaffected side, *i.e.*, a better performance.

#### 3.3.2. Synergy

An ideal shoulder flexion movement can be simply described as the movement whereby an arm rotates around the X-axis in the Y-Z plane (as indicated in Figure 2) with the shoulder as a fulcrum. Therefore, the measured angular velocity in the Y or Z direction can be considered as unnecessary movements resulting from synergy. Based on this thought, the proportion of X-axis angular velocity to the summation of angular velocity in the three axis reflects an assessment indicator for the severity of synergy. The root mean square of angular velocity, *i.e.*, a measure to the magnitude of dynamic angular velocity, is defined as:

(2) $$G_{ij,rms}=\sqrt{\frac{1}{T}\int_{T}G_{ij}{\left(t\right)}^{2}dt}\;,i=1\text{ or }2;\;j=x,y,\;or\;z$$

where $G_{ij}(t)$ denotes a gyro signal, *i* = l or 2 represents the sensor on wrist or elbow, respectively; *j* denotes a specific axis (X, Y, or Z axis). Thus, the indicator for characterizing synergy mentioned as above can be defined as:

(3) $$G_{1,p}=\frac{G_{1x,rms}}{G_{1x,rms}+G_{1y,rms}+G_{1z,rms}}\;G_{2,p}=\frac{G_{2x,rms}}{G_{2x,rms}+G_{2y,rms}+G_{2z,rms}}$$

where $G_{1,p}$ and $G_{2,p}$ denote the assessment indicators on the wrist and elbow, respectively; in general, 0 ≤ G(1,p) ≤ 1 and 0 ≤ G(2,p) ≤ 1. It is expected that a larger indicator value of $G_{1,p}$ or $G_{2,p}$ can be obtained for a post-stroke patient behaving with less synergy and hence better recovery from the stroke.

To give a single index representing the severity of synergy, we further calculate and employ the ratio of the synergy indicators of the affected side to those of unaffected side:

(4) $$G_{r}=\frac{G_{1,p}+G_{2,p}}{G_{1n,p}+G_{2n,p}}$$

where $G_{1n,p}$ and $G_{2n,p}$ denote the synergy indicators on the wrist and elbow of the unaffected side, respectively. This assessment indicator for synergy, *G_r_* (0 ≤ *G_r_* ≤ 1), reflects the clinical evaluation that a larger *G_r_* value closer to unity indicates that the movement of the affected side resembles that of the unaffected side; in contrast, a smaller *G_r_* closer to the null values means a severe synergy.

#### 3.3.3. Movement Performance Speed

For clinical evaluation a shoulder flexion movement that is performed faster and steadily is scored with a higher mark, but only considering the performance time in the absence of information about the total elevation angle may mislead the objectivity of the assessment. In the other word, the movement performance speed conveys a better assessment indicator for the evaluation of stroke recovery. Here, the ratio of affected to unaffected side performance speed, *i.e.*, elevated angle divided by execution time, is proposed as below:

(5) $$T_{r,affected}=\frac{1}{N}\overset{N}{\underset{i=1}{\sum }}\frac{A_{1y,max,i}+1}{t_{affected,i}}\;T_{r,normal}=\frac{1}{M}\overset{M}{\underset{i=1}{\sum }}\frac{A_{1yn,max,i}+1}{t_{normal,i}}$$

where $A_{1y,max,i}$ and $A_{1yn,max,i}$ represent the maximum Y-axis acceleration on the wrist for the affected and unaffected sides, respectively, corresponding to their maximum elevation angle; $t_{affected,i}$ and $t_{normal,i}$ denote the *i*-th movement performance time for the affected and unaffected side, respectively; *N* and *M* are the cycle number of the movement for the affected and unaffected side, respectively. Note that *T_r,affected_* and *T_r,normal_* are actually the performance speed in units of G/s. Therefore, a single indicator for movement performing speed can be defined as:

(6) $$E_{time}=\frac{T_{r,affected}}{T_{r,normal}}$$

For this indicator 0 ≤ E_time_ ≤ 1, so a larger $E_{time}$ corresponds to a better recovery from stroke.

## 4. Results and Discussion

In the study, three indicators were calculated from inertial sensing data, based on the aspects of movement elevation angle, synergy and performance speed, for comparison before and after rehabilitation to indicate the recovery progress of stroke patients. The weights for the three indicators need to be decided to obtain a single objective index that highly correlates the WMFT scores observed from the same patient. The least squares method was applied to estimate the weights of three indicators.

### 4.1. Indicator Analysis

#### 4.1.1. Case 1: Healthy Adult

**Figure 3:**
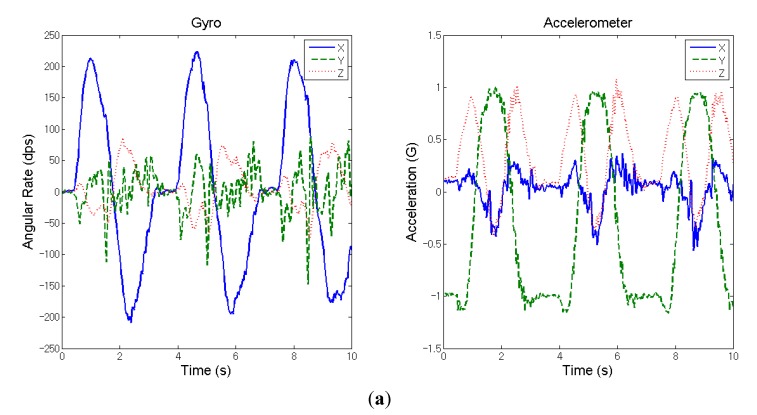

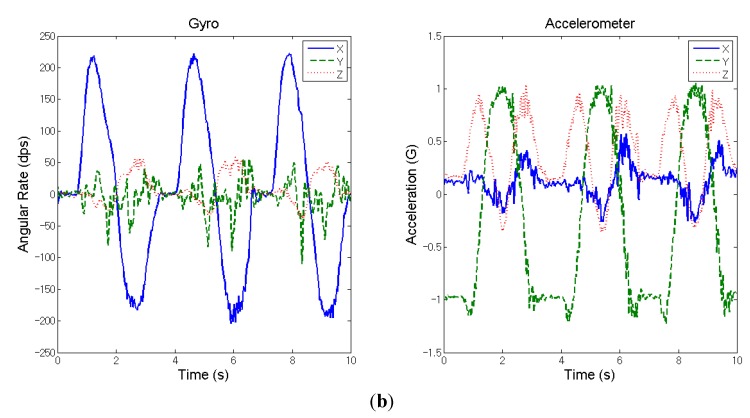
Measured inertial signals from the wrist of a healthy adult during the movement of shoulder flexion (three cycles). (**a**) Dominant hand; (**b**) non-dominant hand.

The test subject in this case is a normal healthy adult without any upper limb impediments. Figure 3 shows the inertial signals measured from his wrist during the shoulder flexion movement. Note that in the subsequent indicator calculation the dominant hand is viewed as the unaffected side whereas the non-dominant hand as the affected side. Table 1 lists the calculated indicators for this case.

**Table 1:** Calculated indicators for Case 1—A healthy adult.

Assessment Indicators	$A_{2,r}$	$G_{r}$	$E_{time}$
Scores	1.00	0.95	0.98

#### 4.1.2. Case 2: Stroke Patient

**Figure 4:**
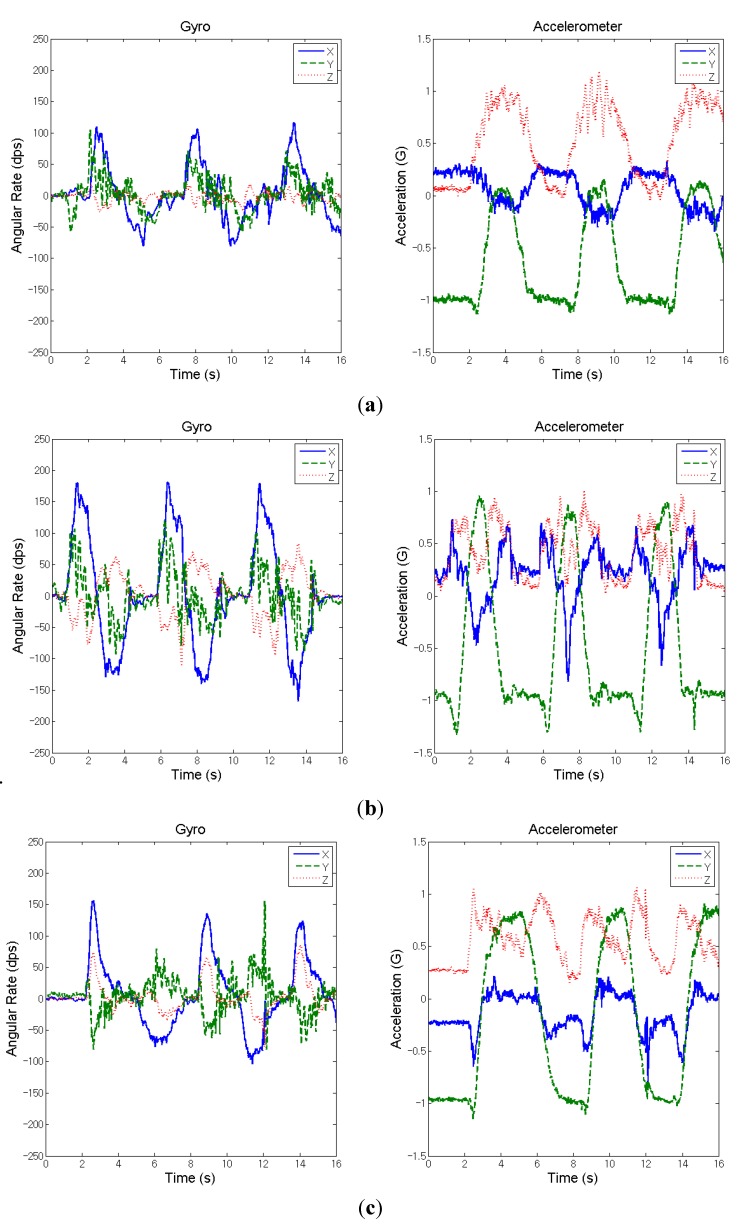
Measured inertial signals from the wrist of a stroke patient during the movement of shoulder flexion (three cycles). (**a**) Affected side (before treatment); (**b**) affected side (after treatment); and (**c**) unaffected side.

The test subject in this case was a 68-year-old man with left-side hemiplegia caused by a right thalamic intra-cerebral stroke. The frequency of treatment is once an hour three times per week and 24 h in total. The partial inertial signals (three cycles) measured on the wrist during the shoulder flexion movement of the affected side are shown in Figures 4a,b for before- and after-treatment, respectively. Figure 4c illustrates the three cycles of inertial data measured from the unaffected side for the calculation of assessment indicators. Table 2 lists the calculated assessment indicators for both before- and after-treatment.

**Table 2:** Calculated indicators for Case 2—A stroke patient.

Assessment Indicators Scores	$A_{2,r}$	$G_{r}$	$E_{time}$
Before-treatment	0.60	0.53	0.67
After-treatment	1.00	0.65	0.88

From the comparison of the indicators for before- and after-treatment, it is noted that in the case the patient’s arm on the affected side can lift as high as the unaffected sided after treatment (the right charts of Figure 4b,c, which is much better than before treatment (the right chart of Figure 4a) as the elevation-angle indicator ***A*_2*,r*_** is only 0.60, as shown in Table 2. On the aspect of angular velocity for synergy, Figure 4b indicates the X-axis angular velocity after treatment is apparently larger than that before treatment (Figure 4a), but the angular velocity in the other two directions still remains to a certain extent; thus, the indicator changed from 0.53 to 0.65 (Table 2), meaning the synergy improvement is not so significant. It is worth noting that the synergy indicator may be overvalued if the unaffected extremity does not perform the movement to the designated position. Overall, in this case the patient actually improved after rehab treatment, as observed from the calculated assessment indicators.

### 4.2. Objective Evaluation Score

At the current stage of the project the IMU data from one healthy adult and eleven patients (both before and after rehab treatment) were collected, and calculated for the assessment indicators; meanwhile, WMFT scores were recorded. The objective assessment measure, *Q*, is defined as below:

(7) $$Q=\left(\alpha \times A_{2,r}+\text{β}\times G_{r}+\text{γ}\times E_{time}\right)\times 100$$

where the indicators $A_{2,r},\;\;G_{r}\text{ and}\;E_{time}$ were defined in Equations (1), (4) and (6), respectively; α, β, and γ denote the weights of indicators. The objective assessment measure *Q* ranges between 0 and 100. The least squares method was applied to determine the weights α, β and γ for the indicators. Therefore, the weights of the three indicators were computed through linear regression between the data sets of objective measure *Q* and subjective WMFT scores. As the summation of weights for the three indicators equals 1, Equation (7) can be rewritten as:

(8) $$Q=100\times \left[\left(1-\text{β}-\text{γ}\right)\right]\times A_{2,r}+\text{β}\times G_{r}+\text{γ}\times E_{time})$$

Thus, we have twenty-three sets of sample data: $\left(A_{2,r,1}\;,\;G_{r,1}\;,\;E_{time,1}\;,\;Q_{1}),\;(A_{2,r,2}\;,\;G_{r,2}\;,\;E_{time,2}\;,\;Q_{2}),\;\ldots ,\text{ and }(A_{2,r,n}\;,\;G_{r,n}\;,\;E_{time,n}\;,\;Q_{n}\right)$, and thus

(9) $$\frac{Q_{i}}{100}=A_{2,r}+\left(G_{r}-A_{2,r}\right)\times \text{β}+\left(E_{time}-A_{2,r}\right)\times \text{γ}+{\text{ε}}_{i}\;,\text{ i}=1,2,\ldots ,\text{n}$$

where ${\text{ε}}_{i}=\frac{Q_{i}}{100}-\frac{{\tilde{Q}}_{i}}{100}$ is the error between the real value and the estimated value, and *n* = 23 here. To solve the linear regression, the least squares method was applied to have:

(10) $$L=\overset{n}{\underset{i=1}{\sum }}{{\text{ε}}_{i}}^{2}=\overset{n}{\underset{i=1}{\sum }}{\left[\frac{Q_{i}}{100}-A_{2,r}-\left(G_{r}-A_{2,r}\right)\times \text{β}-\left(E_{time}-A_{2,r}\right)\times \text{γ}\right]}^{2}$$

Solving β and γ through linear regression for a minimum L results in:

(11) $${\frac{\partial L}{\partial \text{β}}|}_{\hat{\text{β}},\hat{\text{γ}}}=-2\overset{n}{\underset{i=1}{\sum }}\left[\frac{Q_{i}}{100}-A_{2,r}-\left(G_{r}-A_{2,r}\right)\times \hat{\text{β}}-\left(E_{time}-A_{2,r}\right)\times \hat{\text{γ}}\right]\left(G_{r}-A_{2,r}\right)=0$$

(12) $${\frac{\partial L}{\partial \text{γ}}|}_{\hat{\text{β}},\hat{\text{γ}}}=-2\overset{n}{\underset{i=1}{\sum }}\left[\frac{Q_{i}}{100}-A_{2,r}-\left(G_{r}-A_{2,r}\right)\times \hat{\text{β}}-\left(E_{time}-A_{2,r}\right)\times \hat{\text{γ}}\right]\left(E_{time}-A_{2,r}\right)=0$$

Then we obtain

(13) $$\overset{n}{\underset{i=1}{\sum }}A_{2,r}\left(G_{r}-A_{2,r}\right)+\hat{\text{β}}\times \overset{n}{\underset{i=1}{\sum }}{\left(G_{r}-A_{2,r}\right)}^{2}+\hat{\text{γ}}\times \overset{n}{\underset{i=1}{\sum }}\left(E_{time}-A_{2,r}\right)\left(G_{r}-A_{2,r}\right)=\frac{1}{100}\overset{n}{\underset{i=1}{\sum }}Q_{i}\left(G_{r}-A_{2,r}\right)$$

(14) $$\overset{n}{\underset{i=1}{\sum }}A_{2,r}\left(E_{time}-A_{2,r}\right)+\hat{\text{β}}\times \overset{n}{\underset{i=1}{\sum }}\left(G_{r}-A_{2,r}\right)\left(E_{time}-A_{2,r}\right)+\hat{\text{γ}}\times \overset{n}{\underset{i=1}{\sum }}{\left(E_{time}-A_{2,r}\right)}^{2}=\frac{1}{100}\overset{n}{\underset{i=1}{\sum }}Q_{i}\left(E_{time}-A_{2,r}\right)$$

Here the shoulder-related movements including forearm to table, forearm to box, hand to table, hand to box, lift can, and lift basket in WMFT scale scores as $Q_{i}$ value in Equations (13) and (14) were employed. The patients’ scores of shoulder-related movements in WMFT are shown in Table 3. Because of the different scales of the WMFT scale and the objective evaluation score, it is necessary to adjust the WMFT scale using the expression:

(15) $$WMFT_{score}=\frac{WMFT_{raw}}{WMFT_{total}}\times 100$$

where $WMFT_{raw}$ is patient’s score of shoulder-related movements in WMFT, and $WMFT_{total}$ is the total score in WMFT.

**Table 3:** Scores of shoulder-related actions in WMFT for stroke patients.

Case	Pre-Test	Post-Test
**1**	25	24
**2**	9	12
**3**	19	19
**4**	20	22
**5**	20	24
**6**	13	17
**7**	8	15
**8**	20	25
**9**	21	23
**10**	9	12
**11**	11	17

To solve Equations (13) and (14), we substitute three indicators and the adjusted WMFT scores. As a result, the weights of indicators can be acquired. After we have the weights of indicators, the quantitative and objective evaluation score can be rewritten as in Equation (16), and the results are shown in Table 4, where case 12 is the healthy adult.

(16) $$Q=100\times \left(0.20\times A_{2,r}+0.72\times G_{r}+0.08\times E_{time}\right)$$

**Table 4:** Results of objective evaluation score for stroke patients and a healthy adult.

Case	$A_{2,r}$		$G_{r}$		$E_{time}$		Q	
	Pre-test	Post-test	Pre-test	Post-test	Pre-test	Post-test	Pre-test	Post-test
**1**	0.60	1.00	1.00	1.00	0.67	0.88	90	99
**2**	0.39	0.51	0.34	0.42	0.19	0.28	33	42
**3**	0.50	0.63	0.82	0.84	0.28	0.35	71	76
**4**	1.00	1.00	0.58	0.61	0.83	0.83	68	70
**5**	0.34	0.39	0.57	0.63	0.34	0.48	51	57
**6**	0.52	0.69	0.76	0.73	0.48	0.43	69	69
**7**	0.49	0.74	0.41	0.49	0.31	0.60	41	54
**8**	0.48	0.64	0.61	0.64	0.35	0.51	56	63
**9**	0.38	0.59	0.53	0.53	0.20	0.34	47	52
**10**	0.43	0.49	0.38	0.39	0.64	0.68	41	44
**11**	0.43	0.53	0.37	0.41	0.25	0.46	37	44
**12**	1.00		0.95		0.98		96	

**Figure 5:**
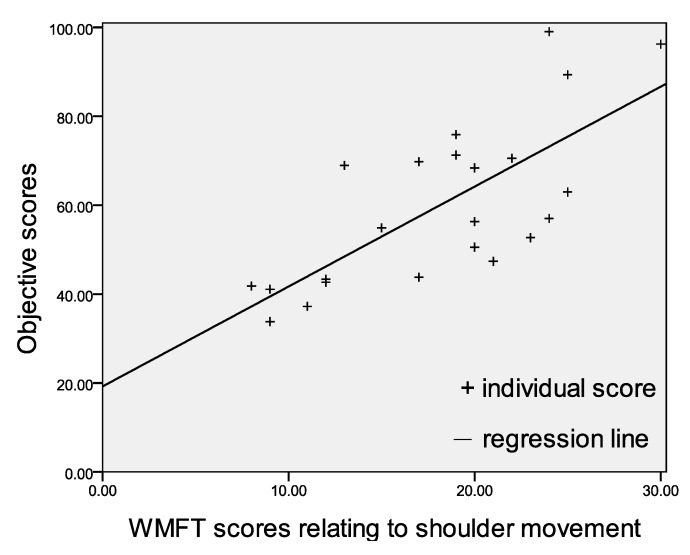
Linear regression between objective scores and WMFT scores relating to shoulder flexion.

To investigate the correlation between the proposed quantitative evaluation method and traditional rehab assessment scales, the scores of objective evaluation and items related to shoulder in WMFT are used to apply linear regression shown in Figure 5. The correlation coefficient *R* equal to 0.72 partly results in part from a lack of enough sample data sets. To improve the objective assessment and be more applicable, more relevant data needs to be collected in the future such that a regression line closer to the scores can be expected.

## 5. Conclusions

In summary, the study implemented for the first time a multiple-channel wireless IMU system that enables one to measure inertial sensing signals from the upper extremities. The proposed objective assessment method appears to have the potential to overcome some of the shortcomings of traditional evaluation methods, and to correctly indicate post-stroke rehab performance. To correlate the objective assessment indicators with the subjective WMFT scales, the study first proposed determining the weights corresponding to each indicator by using the least squares method. The clinical trial results show that more experimental data needs to be acquired to improve the correlation coefficient, or the coefficient of determination.
